# Observations from the Proteomics Bench

**DOI:** 10.3390/proteomes12010006

**Published:** 2024-02-06

**Authors:** Simone König, Karin Schork, Martin Eisenacher

**Affiliations:** 1 IZKF Core Unit Proteomics, University of Münster, 48149 Münster, Germany; 2 Medizinisches Proteom-Center, Medical Faculty, Ruhr-University Bochum, 44801 Bochum, Germany; karin.schork@rub.de (K.S.); martin.eisenacher@rub.de (M.E.); 3 Center for Protein Diagnostics (PRODI), Medical Proteome Analysis, Ruhr-University Bochum, 44801 Bochum, Germany; 4 Core Unit for Bioinformatics (CUBiMed.RUB), Medical Faculty, Ruhr-University Bochum, 44801 Bochum, Germany

**Keywords:** mass spectrometry, bioinformatics, proteomics, false positives

## Abstract

Many challenges in proteomics result from the high-throughput nature of the experiments. This paper first presents pre-analytical problems, which still occur, although the call for standardization in omics has been ongoing for many years. This article also discusses aspects that affect bioinformatic analysis based on three sets of reference data measured with different orbitrap instruments. Despite continuous advances in mass spectrometer technology as well as analysis software, data-set-wise quality control is still necessary, and decoy-based estimation, although challenged by modern instruments, should be utilized. We draw attention to the fact that numerous young researchers perceive proteomics as a mature, readily applicable technology. However, it is important to emphasize that the maximum potential of the technology can only be realized by an educated handling of its limitations.

## 1. Introduction

The journal *Proteomes* is celebrating its 10-year anniversary, and it has become one of the most well-established proteomics journals. When the Joint Editor-In-Chief asked us to contribute to the anniversary issue “with thoughts and reflections of what proteomics has achieved through its history and especially the last ten years”, we did not hesitate very long. The editors wanted “honest opinions that challenge some of the dogma that persists in the proteomics field”, and we do have a number of those. Over the years, several researchers have pointed out problems in proteomics (see [1,2,3] and the references therein), and it is not our goal to repeat them. We would, however, like to present topics and difficulties that we still observe in our daily routines despite many years of great technical advances in the field. To that end, a mass spectrometry (MS) practitioner and bioinformaticians working in the proteomics field joined forces with insights from the analysis of both Q-TOF and orbitrap instrument data. The first researcher was trained by one of the pioneers in biological mass spectrometry, Henry M. Fales (†2010), who shared profound knowledge in both small and large molecule analysis using a wide variety of mass spectrometers. She is now mainly working in protein and peptide analysis across species and methods with a focus on human samples from the associated clinic. The second principal investigator (PI) works with a proteomics facility and supports it with his working group’s software programming and bioinformatics data analysis experience. Our team is not able to offer an opinion on every aspect of proteomics, nor do we present a comprehensive overview; rather, we focus on a few topics we have encountered in our work.

## 2. Results and Discussion—Challenges in Proteomics

### 2.1. Pre-Analytical Issues

Omics researchers are well aware of the need for the standardization of individual experiments as well as the entire project in order to limit, if not eliminate, the variation introduced by sample handling (for exemplary studies, see [4,5,6]). This is easier said than done, especially when multiple laboratories are involved; then, this information is somehow often lost. Parameters such as temperature, sitting times, and centrifugation speed can have considerable influence, as well as personal ambition, skill and technical experience.

#### 2.1.1. Example: Serum Quality

We noted varying serum quality in sample sets of several studies. In one case, only about two-thirds of the samples sent to us were usable for our purpose. Using bradykinin as a reporter peptide [7], we detected a loss of protease activity in these samples compared to all others. We show a photograph of an exemplary 96-well plate of a sample cohort containing some hemolytic samples in Supplementary Figure S1. Clearly, the rupturing of red blood cells with the release of their content into the blood fluid influences the results of subsequent analyses. According to the hemolysis palette published by the Centers for Disease Control and Prevention (CDC) [8] (Supplementary Figure S1), deep orange to red serum samples are not fit for certain analyses like our assay or omics-type experiments. Although instructions to prevent hemolysis are abundantly available from different sources, including the CDC [8], and taught to the medical staff, there still seem to be ample sources for mistakes or reasons for shortcuts.

In this particular sample set, we also detected significant differences in enzyme activity, even for non-hemolytic serum, as a batch effect resulting from the time and place of sampling. That was the more surprising as all collaborating parties were aware of the need for standardization, and precautions had been taken. The reasons for the variation were never properly clarified. In another project, the lower protease activity in some non-hemolytic sera obtained from our clinic was traced back to the random use of different collection tubes by the medical staff.

#### 2.1.2. Example: Tissue Origin

In typical omics projects, two or more groups of samples are compared, e.g., untreated versus treated cells. In cell culture experiments or even in research with laboratory animals, the respective proteomes of each group are well-defined and similar, which is a prerequisite for successful protein abundance analysis. The situation is much more complex in clinical studies because of the large biological variation in patients, even when obvious criteria such as gender and age are taken into account. There are simply too many parameters like nutrition, (self) medication, level of exercise, body weight, genetic predisposition and sanitary conditions to assemble perfect study cohorts. Thus, researchers try to work with large groups in order to alleviate some of the effects. However, for investigations of rare diseases, only a few individuals are available in the first place.

**Figure 1:**
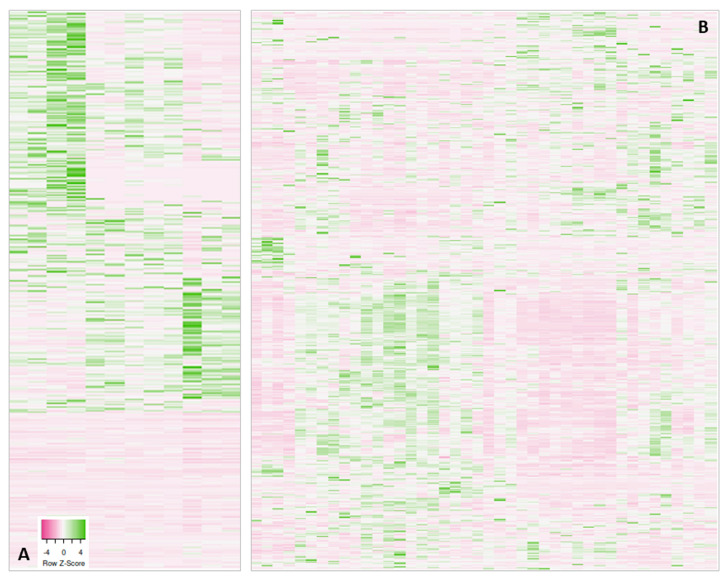
Heatmaps of protein abundancies as detected by MS obtained in two different proteomics projects (samples: x-axis; protein abundancies: y-axis). (**A**) Proteins measured in healthy stomach mucosa used as control versus gastritis and cancer biopsies. (**B**) Serum protein profiles from COVID-19-hospitalized patients. For more information, see references [9,11]. Both examples illustrate the formation of non-uniform sub-groups within the chosen sample cohort. The different coloring originates from different protein abundancies. When a sample group is uniform, the protein abundances should not differ in such a way that green and pink clusters form.

On top of these known limitations, we noted in several clinical studies that the proteomic data of biofluids or tissue samples are of such resolution and quality that individual subgroups can be identified. For instance, in a project working with stomach biopsies, we detected non-uniform protein profiles in the biopsies from the control group of healthy mucosa (Figure 1A) [9]. A double-check with the surgeon revealed that some samples were picked from the corpus rather than the antrum. It has been known, however, since at least the work of Ni et al. [10], that mucosa proteomes differ across the stomach, reflecting regions of different tasks and the presence of specific glands. Conclusively, in order not to distort the results of the investigation, we had to eliminate the corpus samples from the project. On a side note, we also had another problem in that project: in the operating theater, some biopsies had been stored in a different way from the others, promptly resulting in polymer contaminants in these samples, which showed up in the chromatogram; these samples were also lost to the study. Unfortunately, all of these observations were made after complete processing, including MS analysis, had been performed; in fact, the measurements themselves led to questioning of the biopsy quality.

In the second example in Figure 1B, we show serum protein profiles of hospitalized COVID-19 patients [11]. There, it was not too surprising that they differed considerably because the patients were very diverse to begin with; the only unifying parameters were hospitalization and proven COVID-19 infection. Sub-groups could be assembled from these data, which separated moderately from severely and critically ill patients. Again, it was not sensible to use the entire patient group for comparative analyses versus the control groups, but of course, the power of the analysis decreases with the number of subgroups.

### 2.2. Peptide Sequencing

**Figure 2:**
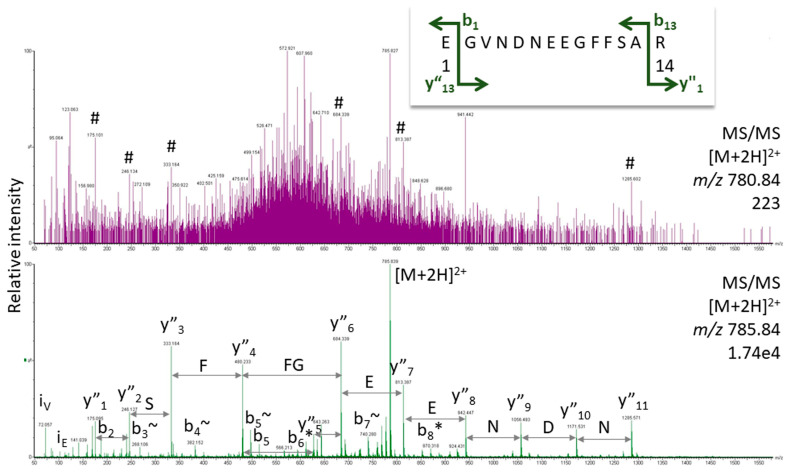
Gas-phase fragmentation spectra for Glu-fibrinopeptide obtained with Q-TOF MS (Synapt G2 Si, Waters Corp., Manchester, UK). In the bottom trace, the instrument was set at the proper isolation mass for the doubly charged peptide (*m*/*z* 785.84). For the top trace, the isolation window was shifted by five mass units to simulate a weak spectrum for illustration purposes. For masses of the expected fragment ions and labels in the bottom spectrum, see Supplementary Figure S2. # indicates fragment ions of Glu-fibrinopeptide at low intensity.

The majority of proteomics studies are based on the so-called bottom–up approach in which the proteins are enzymatically digested before analysis and the peptides are measured. This procedure has several advantages, including the fact that peptides can be measured monoisotopically in the mass spectrometer. Moreover, they can be fragmented in the instrument, and the resulting spectra of fragment ion species are the basis for subsequent protein identification (for a tutorial, see [3]). Although it has been discussed before [2], we need to stress again that only high-quality spectra can provide confident peptide assignments. It is important to be aware that the proteomic experiment measures proteins across an unknown concentration range. Thus, protein matches are reported from abundant proteins as well as from species measured at the detection limit of the instrument. Conclusively, the spectral basis for some protein hits may be excellent (typically for the abundant proteins); for others, however, it may be questionable. For illustration, we show two spectra of Glu-fibrinopeptide—one of great quality showing the entire fragment ion series and another very noisy spectrum with only a few peaks hinting at this peptide (Figure 2). Such low-level data are observed for peptides present only at low concentrations. Modern software is much more successful in finding useful information in these spectra than the human operator, increasing sensitivity. Still, assignments based on poor spectra remain problematic and prone to false positives, especially when they are taken for proof of unique peptides (see below).

Much has been achieved over the past 20 years to improve the capabilities of modern mass spectrometers concerning not only acquisition speed, mass accuracy and reproducibility but also—on a side note—the ease of instrument handling and maintenance. These factors increased the number of reliable protein hits considerably. Thereby, both data-dependent acquisition (DDA; preselection of peptides for fragmentation based on selected parameters such as charge or intensity) and data-independent acquisition (DIA; no preselection, but automated switching of the collision energy between low and high fragmentation of all incoming ions) methods have their merits. None of them are, however, capable of catching all incoming peptides for technical reasons such as scan speed or ion intensity.

### 2.3. Protein Identification

Proteomics has to battle a more complex protein identification process than genomics because transcriptional and translational processes lead to many more protein forms than would be expected from the genetic blueprint. Isoforms or variants originate from single genes as a result of processes like alternative splicing and variable promoter usage [12,13]. They are highly similar in sequence, but only some isoforms may have unique functions. Although most important, those proteins may be present at comparatively low concentrations in a total proteome digest. In order to illustrate this situation, we performed a BLAST analysis of the human tubulin α-1A chain (Uniprot.org, accessed on 21 December 2023) and showed the sequences with more than 90% identity (Supplementary Figures S3 and S4). Exemplarily, three tryptic peptides have been marked, which would need to be detected with good quality in order to verify a certain isoform.

As a result of the huge complexity in measurements of total proteome digests, it is not always possible to find all the unique peptides that would distinguish one protein form from another. Therefore, search algorithms collect all accession numbers for protein sequences that are detected. Consequently, with this global, unbiased experimental approach, isoform analysis is severely limited. As a result of the protein grouping phenomenon (as well as always, spectral quality), we encountered absurd situations such as not finding abundant proteins, which were clearly there, and, on the other hand, finding proteins that were definitely not present. Therefore, the user has always to be aware that, although software is a fantastic tool, it has its limitations.

Protein grouping also influences subsequent gene ontology or pathway analyses. If the protein results table contains several accession numbers for a single protein hit, it is unclear which protein to use for further consideration. Thus, typically, the first match or a preferred hit is chosen, and all others are ignored. Obviously, such analysis delivers only superficial information because it is not carried out based on validated protein forms. Nevertheless, it may be helpful for hypothesis generation.

Of course, the quality of the database available for protein identification is very important. A database can be highly reliable for completely sequenced species but questionable for species with only a few entries. This is often the case for plants, insects or, in general, species that are not as widely researched as humans, mice or rats. Scientists try to circumvent the problem by using the database of a higher-order species with more entries, but this tends to confuse the protein output by the presence of multiple forms of the same protein from different sub-species. These forms may not be easily recognizable as identical or very similar proteins as a result of different protein names. Furthermore, the true protein sequence of the species under investigation will likely be similar but not identical to that of related species. Thus, quantitation efforts in such situations are very problematic.

### 2.4. Modifications

Adding to the complexity of the proteome are the more than 300 known post-translational modifications like glycosylation and phosphorylation, a fact which is summarized with the term “proteoform” [14,15]. Functionally relevant modifications are often sub-stoichiometric, so they may be hard to detect reliably in total proteome analyses. It is, thus, often sensible to enrich the modified proteoforms or, alternatively, deplete the matrix. Spectra of modified peptides should contain most of the expected fragment ions of the b- and y-fragment ion series for confident assignment; low-level data, as shown in Figure 2, are not acceptable as proof for the presence of a certain peptide; they can only serve as an indication that the peptide may be potentially there and that, with enrichment, spectral quality may improve.

Attention also has to be paid to the fact that the electrospray ionization process may introduce oxidative changes on the analyte [16,17] (for an example from phosphorylation analysis, see reference [3]); the search algorithm will not be able to distinguish those artificial modifications from endogenous ones requiring further validation.

Modifications may complicate peptide sequence analysis considerably, and it is not wise to simply allow all possible mass changes in a search algorithm. This measure would only blow up the search space and unduly increase the number of false-positive assignments. Databases for mass changes and peptide modifications such as Unimod and Delta Mass help the analytical chemists with spectral assignments [18,19].

### 2.5. Bioinformatics/Data Analysis Issues

Proteomics based on high-resolution MS is a highly digitalized discipline. It is mandatory to analyze the hundreds of thousands of spectra of a study in silico. Bioinformatics steps should, however, not be misunderstood as post-experiment salvation of suboptimal study design or sample quality; they are rather inherent steps of the workflow that can only properly deal with data obtained in established standardized experiments of a guaranteed quality level. Many authors have worked on the improvement of MS workflows (for examples, see references [20,21,22]). Here, we discuss the way in which we control our experiments.

#### 2.5.1. Quality Assessment/Experiment Reproducibility

Proteomics research requires complex sample preparation and instrumental setups. Reporting all steps of the workflow, as well as quality assessment, has become more and more important since the mid-2000s [23,24]. During the following decade, diverse metrics and tools for quality assessment have been proposed [25,26,27]. In silico quality assessment of experimental data is, first and foremost, a relative evaluation of the parameters of the dataset (e.g., comparison of the detected number of peptide ions, charge states or peptide intensities per run). In this way, outliers within a dataset can be identified. Moreover, if a known reference sample is routinely injected every n runs into the instrument, the comparison of results with those obtained with a new or cleaned instrument may deliver information about increasing contamination of the mass spectrometer source and its internal parts or decreasing quality of the liquid chromatography (LC) column with its lifetime.

**Figure 3:**
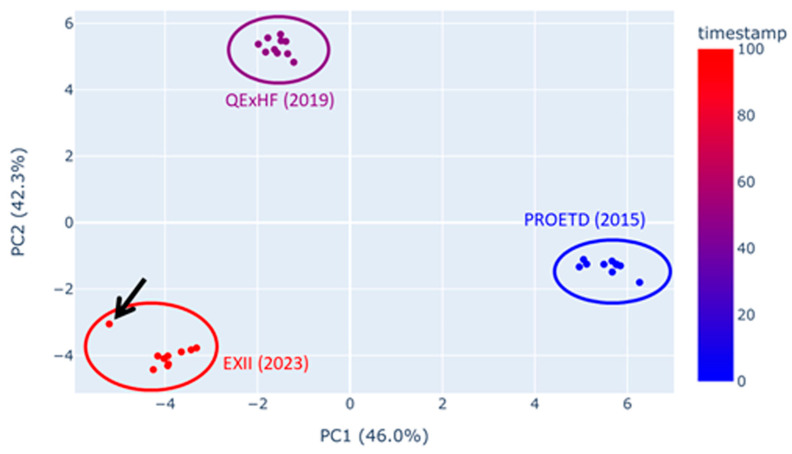
Scatter plot of the first two principal components (PCA-plot) calculated using the QC metrics of the raw data (prior to peptide identification). Each data point represents one raw file. The color gradient is determined by the timestamp, which also indicates the year and machine. The arrow points to an outlier in the EXII raw files.

There was hope among proteomics researchers that with progress in mass spectrometer accuracy and resolution, the technical reproducibility of measurements would improve. In order to test this hypothesis, we here assessed measurements obtained with the in-house prepared standard peptide sample ISA, which is routinely used in our laboratory, which were stored with the sample raw files of each individual study. To that end, we used a quality control (QC) tool that we are developing in order to validate study coherence. It is based on the evaluation of many characteristics of the runs. The QC tool calculates dozens of summarizing values per experiment that characterize the run (e.g., the number of MS2 spectra or the fraction of doubly charged pre-cursors). Some of those values are connected to certain workflow steps (e.g., fractions of identified peptides with missed cleavage sites or quantiles of the total ion chromatogram (TIC) to describe TIC shapes). We applied the tool to 10 consecutive ISA runs, each recorded with three different orbitrap mass spectrometers from Thermo Fisher (datasets labeled as PROETD (2015), QExHF (2019) and EXII (2023)). Principal component analysis (PCA) of all the available quality features has been performed (Figure 3). The technical variance within the ISA runs of each machine is similar (see diameter of dot clouds in Figure 3). The results obtained with the three instruments differ, however, from each other, which is not surprising as their technical parameters vary. There is one outlier in the EXII (2023) runs (indicated in Figure 3 by an arrow). It originated from one run showing heavy deviation at late retention times, most likely resulting from column contamination (e.g., samples containing CHAPS detergent would generate similar ion chromatograms; see Supplementary Figure S5). Moreover, the dot clouds in the PCA show a linear trend, suggesting a gradual change in measurement quality due to column aging or source contamination. Furthermore, technical reproducibility within a dataset does not seem to have improved over the last eight years. The progress in mass spectrometer parameters has not considerably impacted data variability, which is not surprising because a lot of the experimental variation originates from pre-analytical factors (see above) or, as in the case shown here, from contamination.

#### 2.5.2. False-Discovery Estimation with Decoy Sequences

After the Human Genome Project manual spectrum identification was widely replaced by sequence database-driven spectrum identification, it was instantly clear that a race for the number of identified peptide spectrum matches (PSMs) alone was not sufficient or appropriate. For each pair of measured spectrum and theoretical spectrum of a tryptic database peptide, a score was calculated. Thresholding the good from the bad scores was mandatory; this can still be achieved score-system-agnostic with a target-decoy approach [28,29] and a local false-discovery rate (FDR) estimation at each position of the score-sorted peptide spectrum results list. This method is useful and part of state-of-the-art software suites, albeit not aggressively promoted. Moreover, target-decoy implementations face challenges when they fail to generate a sufficient number of decoys to account for the highly accurate pre-cursor masses [30] or when they do not meet other crucial assumptions, such as similar properties of decoy PSMs and incorrect target PSMs [31].

**Figure 4:**
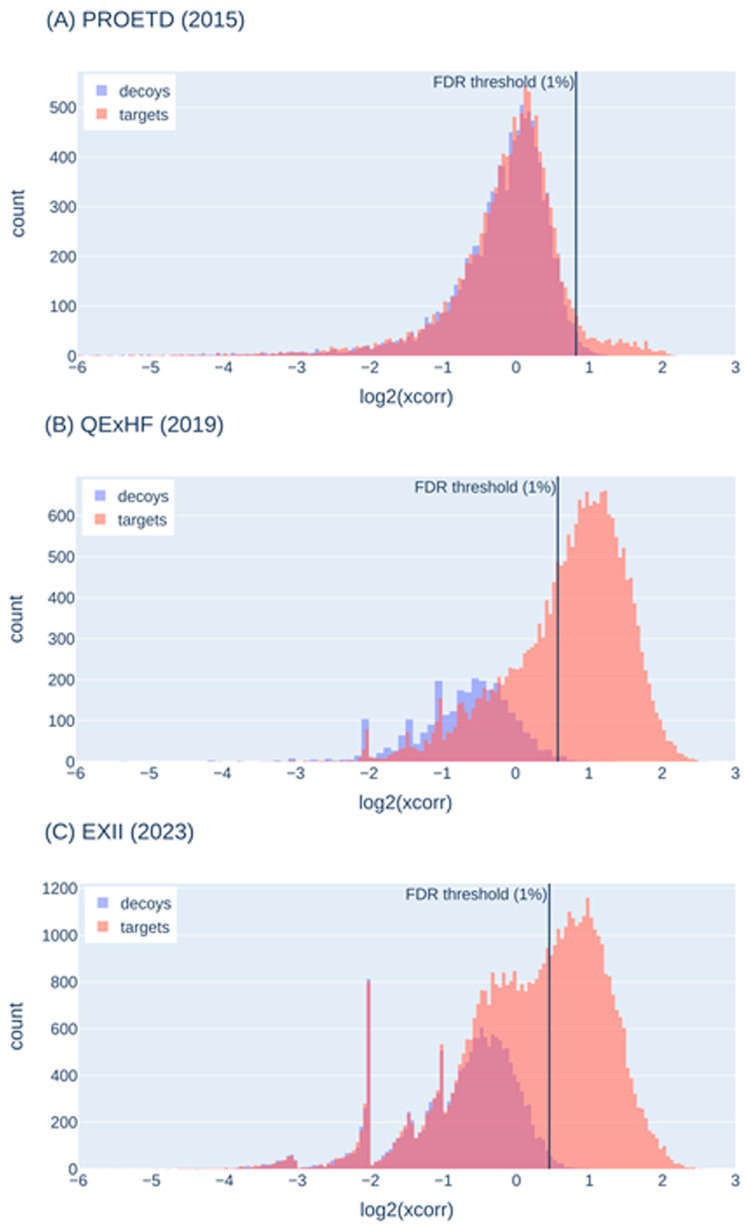
Score histograms for decoy and target PSMs; representative runs selected from PROETD (2015), QExHF (2019) and EXII (2023). The x-axis was cut at −6. The vertical line shows the score threshold for 1% FDR calculated from the respective decoy score distributions.

We visualized the target-decoy behavior on representative runs of the three ISA samples to characterize the target-decoy behavior in our experiments (Figure 4). The decoy PSMs (with their score) represent false-positive target matches around that score so that we can distinguish true from false-positive target scores. Usually, a FDR threshold of 1% is applied, i.e., all target scores higher than this threshold are accepted (shown as a black vertical line in Figure 4).

In the ISA dataset PROETD (2015), the false-positive peak is rather high (targets and decoys nearly perfectly overlaid) and vastly exceeds the peak of true positives (between 1 and 2 on the x axis). In 2023, the peak for true positives (target scores) is higher than in 2019 and higher than the peak for false-positive targets (peak to the left of the threshold). The peak for the false-positive targets is accompanied by a peak for the false-positive decoys, but they do not perfectly overlay, which hints at problems with the decoy assumption (the number of decoy sequences offered to low-quality spectra may not be high enough). The target-decoy histograms of QExHF (2018) indicate even larger problems with the decoy assumption.

In summary, the decoy approach is helpful in separating good from poor matches. With the most recent machines, more true positives than with older instruments and more true positives than false positives are identified so that the disadvantage of not having a perfect separation between good and poor matches is partially compensated. The observed behavior also allows the use of a more stringent FDR (0.01, 0.001 or even lower) without losing too many true-positive hits. Nevertheless, bioinformatic groups are already working on addressing the false-positive detection for state-of-the-art instruments, e.g., by spectrum-specific decoy generation [31].

## 3. Materials and Methods

### 3.1. Description of ISA Files

At the Medizinisches Proteom Center (MPC), ISA is used for regular QC and the monitoring of the performance of the LC-MS setup. The generation of ISA samples is described in Supplementary Materials S1 in detail [32,33,34]. The sample consists of A549 cell culture with six spiked-in peptides. After 15–20 analyses, one ISA is measured for QC; between all runs + ISA, blanks are run to reduce take-over.

Since 2015, the MPC archives all measured raw files. We used files from the most popular mass spectrometers in the years 2015, 2019 and 2023 (Table 1) and compared the results of ten ISA runs, each of which followed at least ten other analyses. We avoided selecting files from times of instrument repair or calibration when ISAs were measured more frequently. We chose consecutive raw files, meaning that no other ISA samples were measured in between the chosen raw files. Details on the MS analysis of the ISA samples are given in Supplementary Materials S2 [35].

### 3.2. QC Tool

For calculating and visualizing the quality measures of the different raw files, we used an updated version of the MaCProQC tool programmed by us [27]. The workflow was re-written in Python 3.8 [36] and is executed via Nextflow [37]. The tool calculates different QC metrics, many of which are based on the paper by Bittremieux et al. [38]. In contrast to the earlier version of the tool [27], the new workflow uses Comet version v2022.01.0 [39] as a search engine instead of Mascot. For feature finding, the FeatureFinderCentroided from OpenMS version 2.8.0 [40] is used. In brief, the workflow first extracts quality metrics directly from the raw files (e.g., number of MS1 and MS2 spectra). Different quartiles are calculated based on the TIC or number of MS events [38]. Then, a peptide identification with Comet is performed. Shared peptides create protein ambiguity, and the necessary reporting of protein groups, potentially including multiple protein accessions (protein inference), is done by PIA version 1.4.8 [41,42]. This results in metrics like the number of PSMs, peptides and protein groups, as well as the distribution of charge states and missed cleavages for the PSMs. The feature detection then reports the number of features and identified features. These metrics are subsequently used for PCA and visualized using the Plotly Python package (Plotly Technologies Inc., Montreal, QU, Canada) [43]. In Comet, two missed cleavages were allowed, and the five best hits were reported. Variable modification was set as oxidation of methionine, and fixed modification was set as carbamidomethylation of cysteine. The tolerance values were chosen depending on the instrument as shown in Table 2. For feature finding, a mass trace *m*/*z*-tolerance of 0.02 and an isotopic pattern *m*/*z*-tolerance of 0.04 were used for PROETD (low-resolution settings), and mass trace *m*/*z*-tolerance of 0.004 and an isotopic pattern *m*/*z*-tolerance of 0.005 were used for EXII and QExHF (high-resolution settings).

### 3.3. Description of Decoy Workflow

The 30 raw files were searched with the Comet search engine, version v2022.01.0 [39]. The FASTA database used for analysis (81,843 entries) contained the human reference proteome version 2022_05 from UniProt as well as the six spike-in peptides (GEPAAAAAPEAGASPVEK, NLVVGDETTSSLR, LQPGDIGIYR, VVVLPSGALQISR, YPGAYYIFQIK, NIPTVNENLENYYLEVNQLEK). Decoys were generated by reversing the original protein sequences within Comet. Only the best hit was reported by Comet, and all other settings were kept the same. The text output file from Comet was further processed with Python scripts. A PSM was counted as a target if it matched with at least one target sequence. If all matched sequences were decoys, it was counted as a decoy. The xcorr scores from Comet were log2-transformed, plotted in histograms and colored by type (targets or decoys) to compare the score distributions between targets and decoys using Plotly [43].

## 4. Conclusions

Many challenges in the field of bioinformatics for proteomics are due to the high-throughput nature of the experiments. Unambiguous protein assignment is complicated by shared peptides [44,45]. Peptide measurement and, subsequently, protein quantification are influenced by suppression effects from the analytical matrix as well as the physico-chemical properties of the individual peptides, namely their ionization efficiency. We have summarized some of the observed problems and their solutions in Table 3.

For an accurate assessment of identification and quantification algorithms, there is a strong need for a standard peptide sample [46] with thousands of potentially shared tryptic peptides in known quantities. However, it is a challenge to experimentally express proteins in correctly known amounts. Even with the use of peptide synthesizers, there are several practical hurdles to generating properly characterized reference samples. Once a highly complex peptide references standard becomes available, sample concentrations could be directly calculated from detector signals by measurement-specific “correction coefficients”. It would help enormously to assess not only the decoy but also the quantitative algorithms. Despite continuous advances in mass spectrometer technology as well as analysis software, there is still a need for dataset-wise QC. With a continuously measured peptide mixture, QC can go beyond one dataset. Work has already begun on the stability of proteotypic peptides [47] used for quantification. False-discovery detection and avoidance is also necessary. The traditional methods are working, but bioinformatics groups are developing better-suited approaches, such as spectra-wise decoys.

The best bioinformatician cannot rescue poor experiments. Our runs with reference samples demonstrate that sample preparation remains the major factor in measurement variability and cannot be compensated by highly advanced technology. Standardization of pre-analytical procedures and proper experimental design remain the most important factors in omics studies.

## Figures and Tables

**Table 1:** MPC mass spectrometers used for this study.

Year	2015	2019	2023
Instrument code	PROETD	QExHF	EXII
Nanoelectrospray mass spectrometer (Thermo Fisher)	Velos Plus	Q Excactive HF	Orbitrap Exploris 480
Analyzer	radial ejection linear ion trap	quadrupole	quadrupole
Detector	electron multiplier	inductive detector	inductive detector
Number of raw files	3172	1930	3287

**Table 2:** Search parameters used in the Comet search engine for the different mass spectrometers.

Project Code	Tolerance		
	Peptide Mass	Fragment Bin	Fragment Bin Offset
PROETD (2015)	10 ppm	1.0005	0.4
QExHF (2019)	10 ppm	0.02	0
EXII (2023)	5 ppm	0.02	0

**Table 3:** Problems in proteomics and their solutions. AC—analytical chemist; SOP—standard operating procedure.

Observed	Solution
**Pre-analysis**	
Poor project design, underpowered analyses, poor group assembly	Communication between PI, collaborators (e.g., surgeons) and AC; joint agreement on study design and sample requirements (SOPs)
Exaggerated expectations on the possible outcome of the analysis when searching for a particular target	Prior to analysis, literature and database searches are made for the known parameters of the target, like its concentration in the cell or its physico-chemical properties. Plausibility consideration: Can the target be expected to be detected with this method at all based on the instrumentation dynamic range and measurement principle? Consideration of enrichment or depletion steps
Lack of standardized sampling, information loss in large projects with many collaborators, free interpretation of SOPs, varying use of consumables and chemicals from different vendors within a project, use of devices such as centrifuges arbitrarily, accidental contamination of labware by colleagues	AC: Training of the PI in sample requirementsPI: Communication of this information to the project group and enforcement of SOPs, proper training of all involved personnel
**Analysis**	
Unexpected difficulties due to sample quality, unknown protein concentration, matrix issues	Running of test samples and adjustment of procedure
Outliers identified in QC visualization (e.g., TIC, PCA) or with QC software	Routine control of the instrument setup with benchmarks to ensure that it is working properly and that outliers result from the sample quality. Instrument maintenance and re-measurement
**Post-analysis**	
Visualization of the protein abundancies (heatmaps) within a project shows distinct sample subgroups	Search for reasons, possible re-measurement or removal of outlier samples, data re-analysis considering subgroups
Hypothesis generation based on weak spectra	Conservative use of the results output from automatic experiments, no undue trust in software-generated Excel tables. Every person working in proteomics should be able to analyze peptide fragment ion spectra manually to have the knowledge to evaluate results from software algorithms. Manually re-check spectral data for potentially important proteins. Familiarization with the meaning of the peptide score in automatic experiments by running target MS/MS on the same peptides in the same sample.
Not a single protein form is assigned, but rather several proteins or isoforms sharing peptide sequences.	Unless a certain protein of interest is separately validated, conclusions should be carefully phrased. The truly functional protein (e.g., modified protein or isoform) may be present sub-stoichiometrically and individually designed analyses may be required.
Overlooked artifacts, e.g., from sample preparation or the analytical process	Careful manual interrogation of spectral evidence, plausibility consideration, validation by orthogonal methods
Excessive use of allowed modifications in database searches, leading to increased false-positive hits	Tailoring of sample preparation and analyses to modification of interest such as phosphorylation (e.g., enrichment), validation
Use of inappropriate databases for analysis of minor species	Plausibility consideration: Is it at all possible that proteins of species can be detected in the bulk sample?
Use of databases of related species in case of unsequenced species	Awareness that the analyte protein will have a (slightly) differing sequence; validation of the peptides by target MS/MS, Edman or other methods
Not finding abundantly present proteins or finding proteins that are not present	Possible software issues; tests of database, algorithm, and search parameters
Decoy histogram does not show sufficient decoys for representation of false-positive target identifications	Optimization of MS parameters (e.g., collision energy) and search engine parameters (e.g., mass tolerances), change of search engine, variation of the protein sequence database (not too large or too small)

## Data Availability

The MS proteomics data have been deposited in the ProteomeXchange Consortium via the PRIDE partner repository with the dataset identifiers PXD048836, PXD048839 and PXD048840. The experimental metadata have been generated using lesSDRF.

## Supplementary Materials

The following supporting information can be downloaded at https://www.mdpi.com/article/10.3390/proteomes12010006/s1. Supplementary figures and experimental details of reference measurements: Supplement.doc.
