# Supplementary material for: Observations from the Proteomics Bench
Source: Proteomes. 2024 Feb 6;12(1):6. doi: 10.3390/proteomes12010006 (PMC10885119; doi:10.3390/proteomes12010006)
Supplement: Supplementary file 1 [file proteomes-12-00006-s001.zip › proteomes-2822180-supplementary.pdf]

proteomes

Communication

Observations from the proteomics bench

Simone König [1,*], Karin Schork [2,3,4] and Martin Eisenacher [2,3,4]

1 IZKF Core Unit Proteomics, University of Münster, 48149 Münster, Germany; koenigs@uni-muenster.de (S.K.)
2 Medizinisches Proteom-Center, Medical Faculty, Ruhr-University Bochum, 44801 Bochum, Germany
3 Center for Protein Diagnostics (PRODI), Medical Proteome Analysis, Ruhr-University Bochum, 44801 Bochum, Germany
4 Core Unit for Bioinformatics (CUBiMed.RUB), Medical Faculty, Ruhr University Bochum
* Correspondence: koenigs@uni-muenster.de; Tel.: 0049-251 8357164

Citation: To be added by editorial staff during production.

Academic Editor: Firstname Lastname

Received: date
Revised: date
Accepted: date
Published: date

Copyright: © 2023 by the authors. Submitted for possible open access publication under the terms and conditions of the Creative Commons Attribution (CC BY) license (https://creativecommons.org/licenses/by/4.0/).

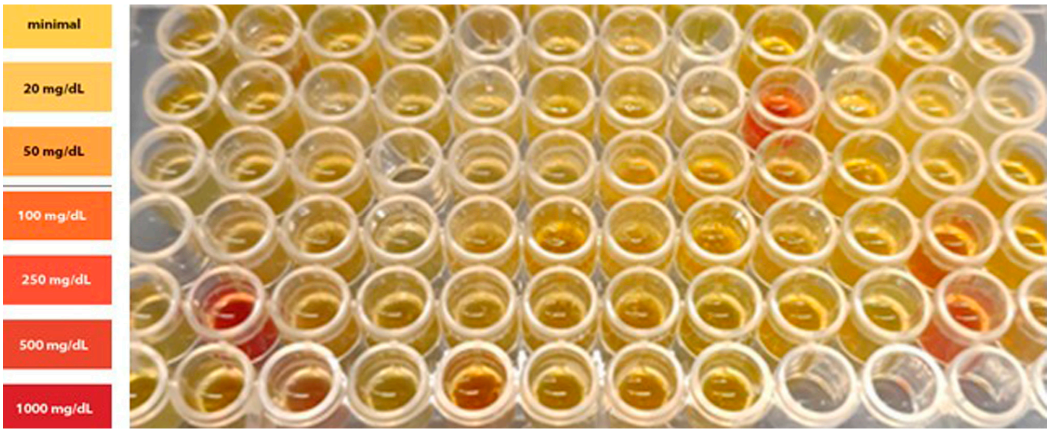

Figure S1. Photograph of a set of serum samples next to the hemolysis palette from the CDC (https://www.cdc.gov/ncezid/dvbd/specimensub/hemolysis-palette.html, accessed Sept. 9, 2023)

b	130.050	187.072	286.140	400.183	515.210	629.253	758.296	887.338	944.360	1091.428	1238.497	1325.529	1396.566	-
i	102.056	30.034	72.081	87.056	88.040	87.056	102.056	102.056	30.034	120.081	120.081	60.045	44.050	129.114
b~	112.040	169.061	268.130	382.173	497.200	611.242	740.285	869.328	926.349	1073.417	1220.486	1307.518	1378.555	-
b*	113.024	170.045	269.114	383.157	498.184	612.227	741.269	870.312	927.333	1074.402	1221.470	1308.502	1379.539	-
	1	2	3	4	5	6	7	8	9	10	11	12	13	14
	Glu	Gly	Val	Asn	Asp	Asn	Glu	Glu	Gly	Phe	Phe	Ser	Ala	Arg
	14	13	12	11	10	9	8	7	6	5	4	3	2	1
y''	-	1441.635	1384.613	1285.545	1171.502	1056.475	942.432	813.389	684.347	627.325	480.257	333.189	246.157	175.119
y~	-	1423.624	1366.603	1267.534	1153.491	1038.464	924.422	795.379	666.336	609.315	462.246	315.178	228.146	157.109
y*	-	1424.608	1367.587	1268.518	1154.475	1039.448	925.406	796.363	667.320	610.299	463.230	316.162	229.130	158.093

Figure S2. Expected fragment ions for Glu-fibrinopeptide as calculated by using MassLynx software (Waters Corp.)

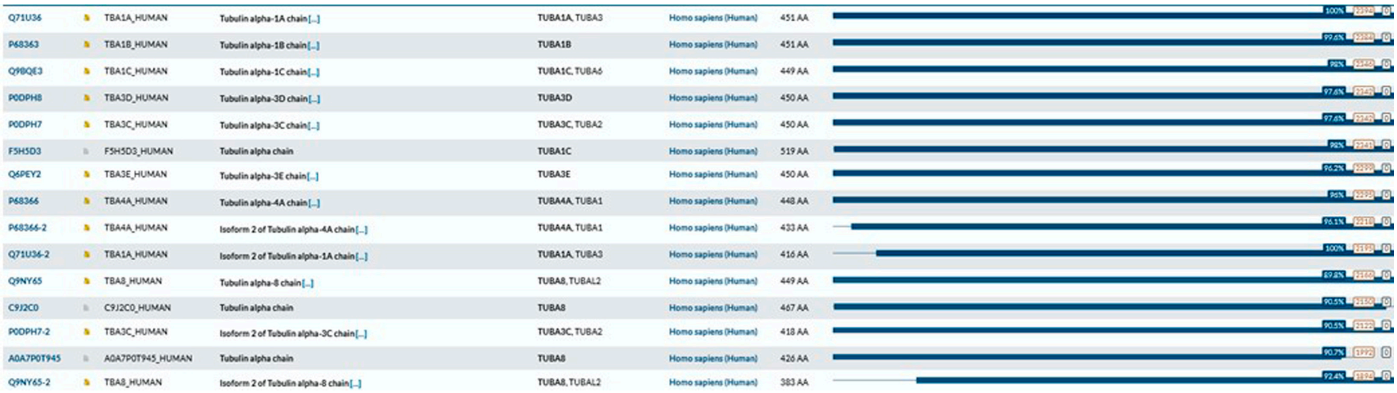

Figure S3. Blast search with human tubulin alpha-1A chain (Q71U36|TBA1A_HUMAN) at Uniprot.org (accessed Sept. 5, 2023). Overview of the top hits.

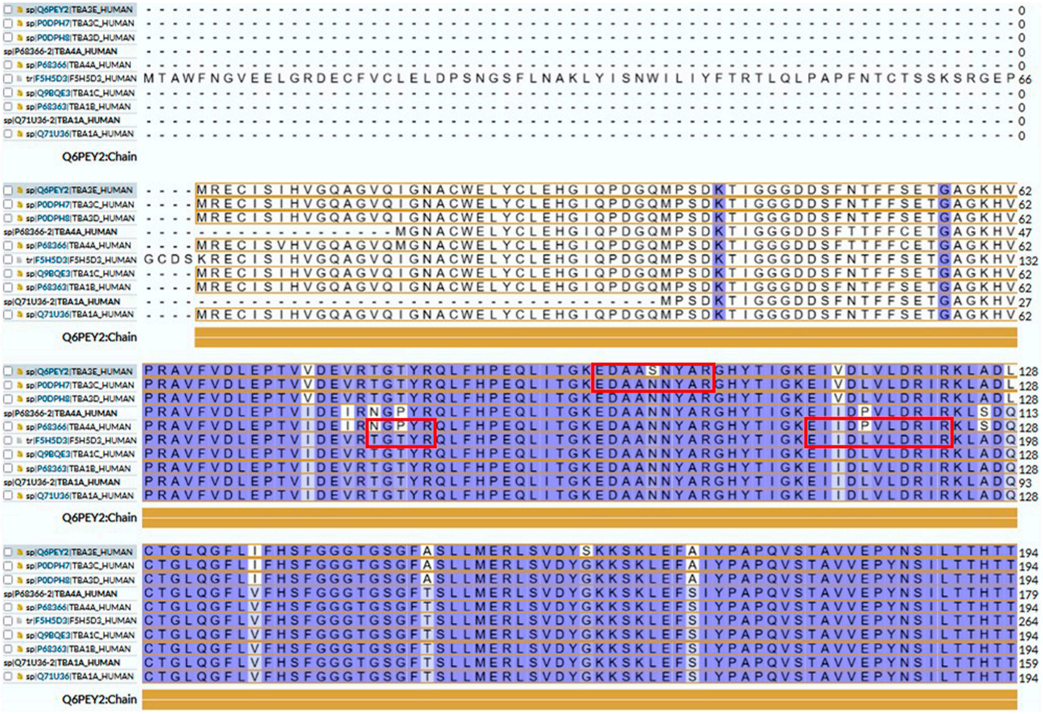

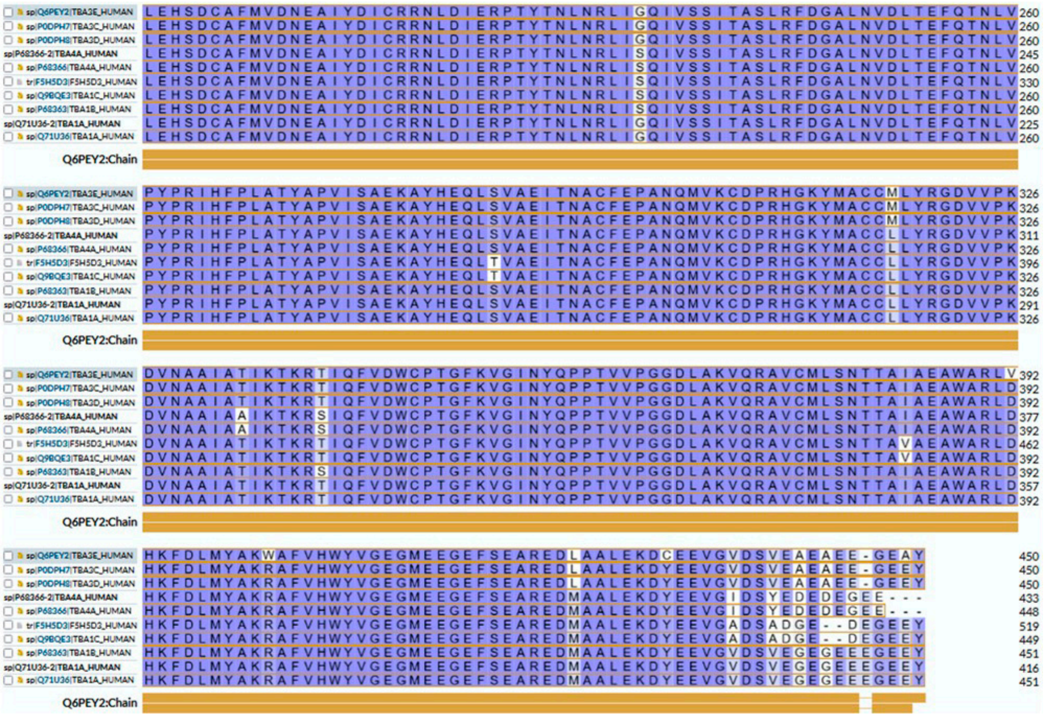

Figure S4. Blast search with human tubulin alpha-1A chain (Q71U36|TBA1A_HUMAN) at Uniprot.org (accessed Sept. 5, 2023). Alignment of the matches better than 90%. Red boxes indicate exemplary unique tryptic peptides.

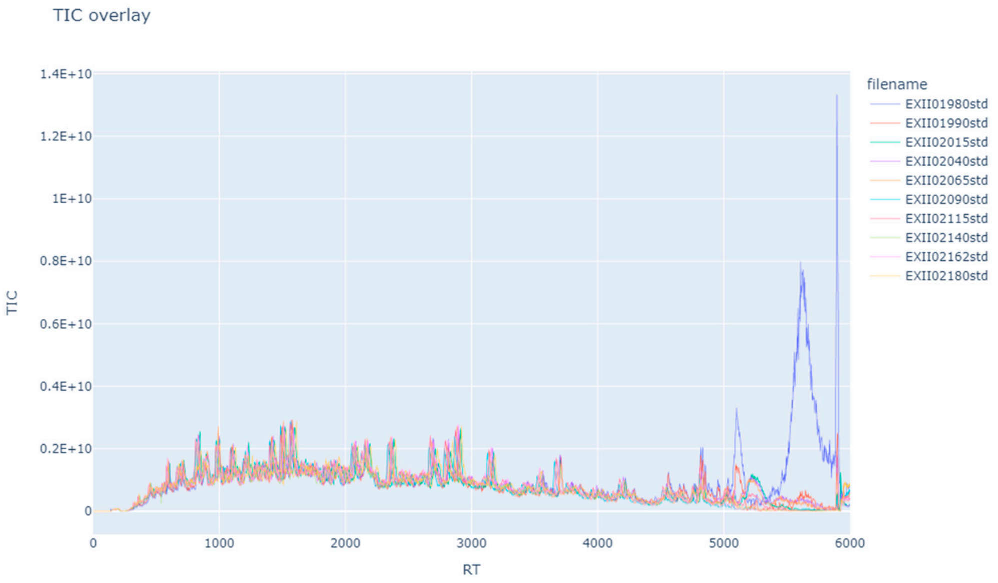

Figure S5. Chromatograms of ten ISA samples measured with the EXII machine (2023). The outlier sample EXII01980std is the blue trace.

Supplementary Methods S1 – Generation of ISA samples

1.1 Cell Culture

Human cancer cell line A549 was purchased from the German Collection of Microorganisms and Cell Cultures GmbH (DSMZ). The frozen cell suspension was thawed at 37 °C and transferred into a 75 cm^2 cell culture flask (Sarstedt AG & Co. KG, Nümbrecht, Germany) containing Dulbecco Modified Eagle medium with 10 % fetal calf serum (both PAN-Biotech GmbH, Aidenbach, Germany). Cell cultures were maintained in a CO_2 incubator at 37 °C and 5% CO_2 until they reached 80% confluence. To obtain sufficient cellular material, cells were expanded by stepwise splitting them into flasks with bigger growth area (175 cm^2) (in total, 49 flasks), using Trypsin/EDTA (PAN-Biotech GmbH). A549 cells were washed with PBS without magnesium and calcium (PBS, PAN-Biotech GmbH) and harvested by mechanical scraping. The cell suspension was transferred into a 50 ml-falcon, washed with PBS and centrifuged at 300 × g for 5 min at room temperature. The cell pellets were stored at -80 °C.

1.2 Cell Lysis and Protein Extraction

The pellets were resuspended in 1.5-fold amount (w/v) of urea buffer (7 M urea, 2 M thiourea, 30 mM tris base, 0.1% sodium deoxycholate) and vortexed. Ultrasound-assisted extraction of proteins was performed using an ultrasonic probe (Bandelin electronics, Berlin, Germany) with 3 cycles of 10 s irradiation (power ~60%) followed by 1 min in silent conditions on ice. To remove the cell debris, the lysate was centrifuged for 15 min at 13.200 rpm at 10°C and the supernatant was transferred into a new tube. The protein concentration was determined according to Bradford [45] using Bio-Rad Protein Assay Dye Reagent (Bio-Rad Laboratories, USA, Cat# 5000006).

1.3 Cell Protein Digestion

500 µg of cell protein were taken for digestion (in total, 38 replicates). 50 mM ammonium bicarbonate was added to reduce the urea concentration to 1 M. Proteins were reduced using dithiothreitol (final 5 mM, 20 min, 56 °C) and alkylated with iodoacetamide (final 15 mM, 20 min, room temperature, in dark). For proteolytic digestion, samples were incubated with trypsin (SERVA Electrophoresis GmbH, Heidelberg, Germany) in an enzyme-to-protein ratio of 1:50 overnight at 37 °C. The digestion was stopped by adding 10 % trifluoroacetic acid (TFA, final 0.1%).

1.4 Sample Quality Control, Aliquoting and Storage

The quality of digestion in each replicate was tested by using LC-MS/MS. Prior, the peptide concentration in each replicate was determined by amino acid analysis [46,47]. 200 ng were injected to an UltiMate™ 3000 RSLC nano system (Thermo Fisher Scientific Inc., Waltham, MA, USA) using a trap column (Acclaim PepMap 100, 300 µm × 5 mm, C18, 5 µm, 100 Å; flow rate 30 µL/min) and separated on an analytical column (Acclaim PepMap RSLC, 75 µm × 50 cm, nano Viper, C18, 2 µm, 100 Å) by a gradient from 5–30% solvent B over 98 min (solvent A: 0.1% formic acid (FA) in water; solvent B: 0.1% FA, 84% acetonitrile in water; flow rate 400 nL/min; column oven temperature 60 °C). Mass spectrometry analysis was performed using Orbitrap Elite™ and LTQ Velos Pro (Thermo Fisher Scientific, Bremen, Germany). Chromatographic profiles, symmetry, width, and intensity of chromatographic peaks, charge states distribution, number of identifications (proteins, peptides) and the number of missed cleavages were checked for each replicate. The 34 replicates that successfully met the assessment criteria were then pooled. Six pre-selected heavy peptides (GEPAAAAAPEAGASPVEK, NLVVGDETTSSLR, LQPGDIGIYR, VVVLPSGALQISR, YPGAYYIFQIK, NIPTVNENLENYYLEVNQLEK) were

spiked to the pool at 100 fmol of each peptide per 200 ng of the peptide matrix. After vigorous mixing, the peptide matrix was aliquoted (15 µg aliquots), dried in a vacuum concentrator and stored at -80 °C until further use.

Supplementary Methods S2 – Mass Spectrometric Analysis of ISA Samples

The ISA samples analzed in this work have been measured with nano-high performance-liquid chromatography (HPLC) followed by electrospray ionization (ESI)-MS/MS on Thermo Fisher Scientific instruments in 2015, 2019 and 2023; the details are described in the following subsections.

2.1 PROETD

Nano-HPLC-MS/MS was performed on an UltiMate 3000 RSLCnano LC system (Dionex, Idstein, Germany). First, samples were loaded on a trap column (Dionex, 75 µm × 2 cm, particle size 3 µm, pore size 100 Å) with 0.1% TFA (flow rate 10 µl/min). After washing, the trap column was serially connected with an analytical C18 column (Dionex, 75 µm × 25 cm, particle size 2 µm, pore size 100 Å). The peptides were separated with a flow rate of 400 nl/min using the following solvent system: (A) 95% acetonitrile, 0.1% FA; (B) 80% acetonitrile, 0.1% FA. In a first step a gradient from 100% A to 40% B (95 min) was used, followed by a second gradient from 40% B to 95% B within 2 min and finally a gradient from 95% to 5% B. The HPLC system was directly coupled to a nanoelectrospray ionization source (Thermo Fisher Scientific, Bremen, Germany). ESI-MS/MS was performed on a LTQ Orbitrap Velos (Thermo Fisher Scientific). MS spectra were scanned between 300 and 2000 *m*/*z* with a resolution of 30,000 and a maximal acquisition time of 500 ms. The m/z values initiating MS/MS were set on a dynamic exclusion list for 35 s. Lock mass polydimethylcyclosiloxane (*m*/*z* 445.120) was used for internal recalibration. The 10 most intensive ions (charge > 1) were selected for MS/MS-fragmentation in the ion trap. Fragments were generated by low-energy collision-induced dissociation on isolated ions with collision energy of 35% and maximal acquisition time of 50 ms.

2.2 QExHF

Nano HPLC analysis was performed on an UltiMate 3000 RSLC nano LC system (Thermo Fisher Scientific, Bremen, Germany). In detail, samples were loaded on a trap column (Acclaim®PepMap 100, 100 µm × 2 cm, C18, particle size 5 µm, pore size 100 Å) with 0.1% TFA (flow rate 10 µl/min) for 8 min, serially connected to an analytical column (Acclaim®PepMap RSLC, 75 µm x 50 cm, C18, particle size 2 µm, pore size 100 Å). Peptides were separated with a flow rate of 400 nl/min and a solvent gradient from 4% A to 40% B (A: 0.1% FA, B: 84% acetonitrile, 0.1 % FA) for 95 min. Columns were subsequently washed by eluting 95% solvent B for 12 min followed by an equilibration to 5% solvent B for 5 min resulting in a total of 120 min run time. The HPLC system was online-coupled to the nano ESI source of a QExactive HF mass spectrometer (Thermo Fisher Scientific, Bremen, Germany). Ionization was performed with a spray voltage of 1500 V and capillary temperature of 275 °C. In ESI-MS/MS analysis, full MS spectra were acquired in the range from 350 to 1400 *m*/*z* with a resolution of 60.000 at 200 *m*/*z* for the detection of precursor ions (AGC target 3e6, 80 ms maximum injection time). The *m*/*z* values initiating MS/MS were set on a dynamic exclusion list for 30 s, and the 20 most intensive ions (charge state +2, +3, +4) were selected for fragmentation. MS/MS fragments were generated by high-energy collision-induced dissociation (HCD), with a fixed normalized collision energy (NCE) of 28. The fragments were analyzed in an Orbitrap mass analyzer with 60.000 resolution (AGC target 1e6, 120 ms maximum injection time) an isolation window of 1.6 *m*/*z* and a fixed first mass of 100 *m*/*z*. The scan range was set between 200-2000 *m*/*z* and an intensity threshold of 4.2e04 was applied.

2.3 EXII

200 ng of sample peptides in 0.1 % TFA were injected to Vanquish Neo UHPLC system (Thermo Fisher Scientific, Bremen, Germany) using a PepMap Neo C18 Trap Cartridge (300 µm × 0.5 cm, particle size 5 µm) and subsequently separated on an analytical column (DNV PepMapTM Neo, 75 µm x 150 mm, C18, particle size 2 µm, pore size 100 Å). Peptides were separated with a flow rate of 400 nl/min and a solvent gradient from 1% B to 21% B (B: 84 % acetonitrile, 0.1% FA) for 70 min, with a subsequent increase up to 40% for 25 min and washing for 5 min with 95% B. Peptides were ionized by ESI and injected into an Orbitrap Exploris 480 mass spectrometer (Thermo Fisher Scientific, Bremen, Germany). The capillary temperature was set to 275 °C, the spray voltage to 1800 V. Full MS spectra were acquired in the range from 375 to 1500 *m*/*z* with a resolution of 120,000 at 200 *m*/*z* (AGC target 250%, maximum injection time 150 ms). 25 most intensive ions (charge state +2 - +7) were selected for fragmentation, and the MS/MS fragments were generated by HCD, with a fixed NCE of 31. Intensity threshold was set to 1.0e04, dynamic exclusion to 80 s. The fragments were analyzed in an Orbitrap mass analyzer with a 30.000 resolution (isolation window 0.8 m/z, AGC target 150 %, maximum injection time 50 ms).
